# Bacillus Calmette-Guérin as adjuvant platform enhances immunogenicity of conserved epitopes from structural proteins of SARS-CoV-2

**DOI:** 10.3389/fimmu.2026.1775087

**Published:** 2026-07-16

**Authors:** Ana de Souza Santos, Leonardo Pereira de Araújo, Diego Jose Belato Orts, Hernan Hermes Monteiro da Costa, Kely Catarine Matteucci, Evandro Neves Silva, Karen Cristina Oliveira, Giovane Galdino, Carlos Roberto Prudencio, Patrícia Paiva Corsetti, Leonardo Augusto de Almeida

**Affiliations:** 1 Laboratory of Molecular Biology of Microorganisms, Federal University of Alfenas (UNIFAL-MG), Alfenas, MG, Brazil; 2 Laboratory of Immunotechnology, Center of Immunology, Institute Adolfo Lutz, São Paulo, SP, Brazil; 3 Interunit Graduate Program in Biotechnology, University of São Paulo, São Paulo, SP, Brazil; 4 Multipurpose Laboratory, Butantan Institute, São Paulo, SP, Brazil; 5 Department of Biochemistry and Immunology, Ribeirão Preto Medical School, University of São Paulo, Ribeirão Preto, SP, Brazil

**Keywords:** BCG vaccine, conserved epitopes, multi-epitope vaccine, SARS-CoV-2, trained immunity

## Abstract

The development of effective and broadly protective vaccines against SARS-CoV-2 remains a global priority. Conserved epitopes from viral structural proteins (E, M, N, and S) represent promising targets less affected by emerging mutations, while *Bacillus Calmette-Guérin* (BCG) offers unique adjuvant and delivery properties. This study aimed to validate conserved SARS-CoV-2 epitopes in combination with BCG and to design multiepitope vaccine constructs *in silico*. Dot blot assays confirmed recognition of five synthetic peptides by sera from convalescent patients. *In vitro*, BCG–peptide formulations activated the MAPK pathway and induced trained immunity signatures in macrophages. *In vivo*, immunized mice showed modulation of IgG subclasses and increased IL-6, TNF-α, and IFN-γ production. Splenocytes from vaccinated animals secreted high cytokine levels upon restimulation, suggesting memory responses. *In silico* modeling indicated stable, antigenic, and non-allergenic multiepitope constructs with favorable immune simulations. Together, these findings highlight BCG–epitope formulations as promising next-generation vaccine candidates against SARS-CoV-2.

## 1 Introduction

The COVID-19 pandemic, caused by SARS-CoV-2, had an unprecedented global public health impact, with approximately 780 million cases and 7.1 million deaths reported as of August 2025 (1–4). Viral transmission control was achieved through global mass vaccination programs, with coverage rates ranging from 90% to 100% in several countries (1–4). Although there are indications of seasonality for the virus, its pattern is not yet clearly defined, making the future dynamics of circulation uncertain (5, 6). It is estimated that viral containment will depend primarily on maintaining large-scale vaccination campaigns targeting vulnerable groups, such as the elderly and immunocompromised individuals, who are at greater risk of developing severe forms of the disease, requiring hospitalization, and potentially progressing to death (1, 5–7).

Conventional or next-generation vaccines used in COVID-19 control include inactivated virus vaccines (CoronaVac), viral vectors (Oxford–AstraZeneca, Johnson & Johnson), and messenger RNA platforms (Pfizer-BioNTech, Moderna), all of which have shown high efficacy in reducing viral load (8, 9). Most of these vaccines target the Spike (S) protein, the main surface antigen and highly immunogenic (8, 9). However, this protein harbors most emerging mutations, especially in the receptor-binding domain (RBD), which may favor the emergence of variants of concern (VOCs) and compromise vaccine long-term efficacy (10, 11). In contrast, other structural proteins, such as Envelope (E), Membrane (M), and Nucleocapsid (N), have relatively conserved regions with recognized antigenic potential (12–15).

Our group identified, through *in silico* analyses, conserved epitopes in structural proteins (E, M, N, and S) with high potential for MHC binding and activation of CD4^+^ and CD8^+^ T lymphocytes (14). These epitopes showed predicted capacity to induce adaptive responses, with CD8^+^ lymphocytes eliminating infected cells and CD4^+^ lymphocytes assisting antibody production (14). However, alone, these epitopes tend to elicit less robust responses than whole proteins or live attenuated vaccines, making the use of adjuvants necessary to enhance their immunogenicity (15–18).

A promising example is the use of *Bacillus Calmette-Guérin* (BCG) as an adjuvant and delivery platform (19). Originally developed for tuberculosis prevention, BCG exhibits broad immunomodulatory effects, induces trained immunity, and may confer cross-protection against viral infections (19, 20). Additionally, combinations of BCG with SARS-CoV-2-specific antigens, in recombinant form expressing the N or S proteins, have induced robust cellular responses (CD4^+^ and CD8^+^) and high antibody titers (21–23). Evidence also indicates that heterologous association of BCG with peptides holds significant potential, as demonstrated in a study where BCG combined with *Schistosoma mansoni* paramyosin resulted in greater protection against schistosomiasis in mice compared to paramyosin alone (19).

Given this context, the present study aims to evaluate the immune response induced by conserved SARS-CoV-2 epitopes administered with BCG as a heterologous adjuvant, seeking to enhance their presentation to immune cells via MHC and simultaneously induce adaptive immune responses and potential trained innate immunity. For this purpose, *in vitro* and *in vivo* approaches were employed to validate the ability of these epitopes to stimulate both cellular and humoral (antibody-mediated) responses, as well as perform an *in silico* prediction of a potential multi-epitope construct. These findings are expected to contribute to the development of next-generation vaccines capable of providing broader protection against SARS-CoV-2 variants.

## 2 Materials and methods

### 2.1 Synthesis of epitopes

Five synthetic peptides corresponding to immunodominant epitopes of SARS-CoV-2 structural proteins were selected and synthesized. These included epitopes from the envelope (P1), membrane (P2), nucleocapsid (P3), and spike (P4 and P5) proteins (14). The peptides were synthesized by solid-phase peptide synthesis and purified via reverse-phase HPLC, with identity confirmed by mass spectrometry. All procedures were performed by a specialized provider (Aminotech Pesquisa e Desenvolvimento, Sorocaba, SP, Brazil). Analytical data including HPLC chromatograms and mass spectra are available in the **Supplementary Material** (**Supplementary Figure 1**).

### 2.2 Expression and purification of recombinant SARS-CoV-2 receptor binding domain

The recombinant Receptor Binding Domain (RBD) protein was expressed using HEK 293F cells and the ExpiFectamine 293 Transfection Kit (Thermo Fisher, Waltham, USA). Purification was performed on two steps. First, the recombinant RBD was purified by immobilized metal ion affinity chromatography using a HisTrap™Excel columns (GE Healthcare, IL, USA), and then a size-exclusion chromatography using a Superdex™ 75 column (Cytiva Life Sciences, MA, USA) (23).

### 2.3 Study design - selection of serum samples from COVID-19 patients

For this experiment, twenty serum samples from patients tested positive for SARS-CoV-2 infection admitted at Institute of Infectology Emilio Ribas (IIER) between March and June 2020, and from Institute Adolfo Lutz (IAL) routine were enrolled in this study. Clinical symptoms, RT-qPCR confirmation, serostatus verification with in-house (24) and commercial kit SARS-CoV-2 IgG/IgM lateral flow assay (Cellex, NC, USA) were considered control selection criteria. Patients were categorized as asymptomatic, mild, moderate and severe/critical ill according to the worst level of COVID-19 recorded during hospitalization and following the IIER clinical guidelines. Five serum samples collected before September 2019, confirmed negative for SARS-CoV-2 IgG and IgM antibodies, were used as negative controls. Participation was voluntary and participants signed an informed consent under the Ethical Committee (CAAE: 32264120.5.2001.0061).

### 2.4 SARS-CoV-2 peptide dot blot hybridization assay

Five synthetic peptides (P1 to P5, 1 μg of each) were spotted on a high-binding 0.22 µm Hybond^TM^ nitrocellulose membranes (Amersham, UK) using the Bio-Dot Apparatus (Bio-Rad, CA, USA). Recombinant RBD protein (0.5 μg) and bovine serum albumin (BSA, 0.5 μg) were included as positive and negative controls, respectively. Membranes were blocked with TBS supplemented with 5% of skim milk and then incubated with TBS + 2.5% milk containing 1:2500 dilution of each patient serum. Afterwards, membranes were incubated with goat anti-human IgG (whole molecule) HRP conjugated (Sigma, CA, USA). Incubation steps were carried out with gentle agitation for 1 hour at room temperature and membranes were washed 3 times for 5 min with TBS-T (TBS + 0.1% Tween 20 [v/v]) after each step. To ensure the best performance of the dot blot immunoassay, an optimum primary antibody working concentration was determined. 1 μg of each peptide was spotted onto the nitrocellulose membrane and, after the blocking step, membrane strips were incubated with a two-fold serial dilution (from 1:2500 to 1:20,000) of the primary antibody, followed by incubation with a single secondary-antibody dilution (1:8000). The optimal peptide concentration was determined based on standard checkerboard titration procedures. Chemiluminescent reaction was performed using SuperSignal™ West Pico PLUS Chemiluminescent Substrate (Thermo Scientific, MA, USA). Images and densitometry analysis were recorded using iBright CL1500 image system (Thermo Scientific). Densitometry analysis was performed by integrating the levels of pixels surrounded by a circular selection. Density of immunoreactivity dots is reported as arbitrary units (a.u.) and expressed as the mean ± SEM (23, 24).

### 2.5 Animal model and ethical statement

Isogenic female C57BL/6 mice, aged 6 to 8 weeks, were obtained from the Central Animal Facility of the Federal University of Alfenas (UNIFAL-MG, Brazil). Animals were housed under controlled environmental conditions (22 ± 2 °C, 50-60% relative humidity, 12:12-h light/dark cycle) with ad libitum access to water and a standard rodent diet (Nuvilab CR1, Nuvital, Brazil). All experimental procedures were conducted in strict compliance with Brazilian laws regarding animal experimentation (Laws 6.638/1979 and 9.605/1998) and in accordance with the ARRIVE (Animal Research: Reporting of *In Vivo* Experiments) guidelines, as described at https://arriveguidelines.org. The experimental protocol was approved by the Institutional Animal Care and Use Committee (CEUA) of UNIFAL-MG under protocol number 0008-2021.

### 2.6 Experimental animals and bone marrow-derived macrophage culture

Bone marrow cells were isolated from the femurs and tibias of 6–8 week-old C57BL/6 mice euthanized by intraperitoneal administration of a lethal dose of ketamine (0.4 mg/g) and xylazine (0.2 mg/g). The bone marrow was flushed with sterile saline, centrifuged (1200 rpm, 10 min, 4 °C), and resuspended in Dulbecco’s Modified Eagle Medium (DMEM; Gibco, USA) supplemented with 10% heat-inactivated fetal bovine serum (FBS), 1% penicillin–streptomycin, and 1% HEPES buffer. The cell suspension was filtered through a 70 µm nylon mesh to remove debris and aggregates and then plated for 2 h at 37 °C and 5% CO_2_ to allow the adhesion of mature cells. Viable cells were harvested and cultured at a density of 5×10⁵ cells/well in 24-well plates in medium containing 25 ng/mL macrophage colony-stimulating factor (M-CSF). On day 5, the culture medium was replaced with a fresh medium containing 50 ng/mL M-CSF. After 10 days of culture, bone marrow-derived macrophages (BMDMs) were obtained.

BMDMs were stimulated with BCG Moreau strain (MOI 0.1) alone or in combination with peptides P1, P2, P3, P4, or P5 (1 μg/well). Stimulations were performed in two phases: an initial 24-hour stimulation followed by collection of supernatants and medium replacement (DMEM without M-CSF), and a second 48-hour stimulation under the same conditions, after which supernatants were again collected for cytokine analysis (25, 26).

### 2.7 Protein extraction and Western blotting

Total proteins from unstimulated or stimulated BMDMs were extracted by adding 100 μL of RIPA lysis buffer (Sigma-Aldrich, R0278) supplemented with a protease inhibitor cocktail (10%, Sigma-Aldrich, P2714) and a phosphatase inhibitor cocktail (1%, Sigma-Aldrich, P0044) directly to the wells. Lysates were collected, centrifuged at 12,000 rpm for 5 min at 4 °C, and the protein concentration was determined. Samples were mixed with 4× Laemmli buffer (Bio-Rad, 1610747) containing 10% β-mercaptoethanol, then boiled at 98 °C for 5 min. Equal amounts of protein (10 μg) were loaded onto SDS-PAGE gels, electrophoresed (80 V initially, then 120 V for approximately 2 h), and transferred to nitrocellulose membranes.

Membranes were blocked with 5% skim milk in TBS-T for 2 h at room temperature, then incubated overnight at 4 °C with primary antibodies diluted 1:1000 in blocking buffer: anti-p38 MAPK (Cell Signaling Technology, 8690), anti-ERK1/2 (p44/42 MAPK; CST, 4695), and anti-SAPK/JNK (CST, 9252). After washing, membranes were incubated for 2 h with HRP-conjugated secondary antibody (1:2000, CST, 7074). Detection was performed using Clarity Western ECL substrate (Bio-Rad, 1705060), and images were acquired with a ChemiDoc XRS+ system (Bio-Rad) and analyzed using Image Lab software (Bio-Rad). Band intensities were normalized to the loading control (β-actin) and quantified relative to the untreated control (NT) to assess MAPK activation upon stimulation.

### 2.8 Cytokine measurement by ELISA

IL-6, IL-1β, and TNF-α levels were quantified using Mouse IL-6/IL-1β/TNF-α Murine Standard ABTS ELISA Development Kit (PreproTech, Cranbury, NJ, USA), following manufacturer instructions. Briefly, high-binding ELISA plates (Nunc MaxiSorp) were coated overnight at 4 °C with capture antibodies (1 μg/mL). After washing and blocking with 1% BSA in PBS (1h, room temperature), samples and standards were incubated (2h, room temperature), followed by detection antibodies (1 μg/mL, 2h, room temperature), avidin-HRP conjugate (1:200, 30 min), and ABTS substrate. Absorbance was read at 405 nm (650 nm correction) using a microplate reader.

### 2.9 Animal immunization

Female C57BL/6 mice, six per group, were immunized by intramuscular injection with a vaccine formulation containing Mycobacterium bovis-BCG at a concentration of 5×10^4^ colony-forming units, combined with five synthetic peptides derived from structural proteins of SARS-CoV-2. Each animal received a total volume of 50 µL per dose, consisting of 5 µL of BCG suspension, 25 µL of a pooled peptide mixture, corresponding to a total of 25 µg of peptides per dose (5 µg of each peptide at a concentration of 1 μg/μL), and 20 µL of sterile saline solution. Two immunizations were administered with a 15-day interval between doses. Fifteen days after the second immunization, animals were euthanized, and blood and spleen samples were collected for subsequent humoral and cellular immune analyses.

### 2.10 Splenocyte culture

C57BL/6 mice were euthanized by intraperitoneal administration of a lethal dose of ketamine (0.4 mg/g) and xylazine (0.2 mg/g). Spleens were aseptically collected and mechanically dissociated to obtain single-cell suspensions. The suspensions were centrifuged at 1200 rpm for 7 min at 4 °C, and the supernatant was discarded. Red blood cells were lysed using 1 mL of ACK buffer (0.15 M ammonium chloride, 1.0 mM potassium bicarbonate, and 0.1 mM disodium EDTA, pH 7.2). After 5 min of incubation, the volume was completed with sterile saline, followed by centrifugation under the same conditions. The final cell pellets were resuspended in RPMI 1640 medium supplemented with 10% heat-inactivated fetal bovine serum and 1% penicillin–streptomycin. Cell viability was determined by Trypan Blue exclusion, and splenocytes were adjusted to a final concentration of 1×10^6^ viable cells per well.

Cells were seeded in 96-well plates and stimulated for 24 hours under different conditions according to the immunization groups. Stimulation was carried out in a complete culture medium supplemented with 10% fetal bovine serum, 1% penicillin–streptomycin, and 1% HEPES buffer. Each well was individually stimulated with one of five synthetic peptides (P1–P5), derived from SARS-CoV-2 structural proteins, at a final concentration of 1 µg per well. Additional experimental conditions included stimulation with BCG at a multiplicity of infection (MOI) of 0.1, or co-stimulation with BCG in combination with each individual peptide. Following incubation at 37 °C in an atmosphere containing 5% CO_2_, culture supernatants were collected and stored for subsequent cytokine analysis.

### 2.11 Cytokine analysis by CBA

Cytokine production was assessed in splenocyte culture supernatants using the BD Cytometric Bead Array (CBA) Mouse Th1/Th2/Th17 Cytokine Kit (BD Biosciences, Cat. No. 560485). This multiplex flow cytometry–based assay simultaneously quantified the concentrations of interleukin-2 (IL-2), interleukin-4 (IL-4), interleukin-6 (IL-6), interleukin-10 (IL-10), interleukin-17A (IL-17A), interferon-gamma (IFN-γ), and tumor necrosis factor (TNF). Samples were prepared, stained, and acquired on a calibrated flow cytometer according to standard procedures provided in the kit manual. Data were analyzed using FCAP Array software, and cytokine concentrations were determined by interpolation from standard curves constructed for each analyte.

### 2.12 Serological assay for total IgG and subclasses

To evaluate the humoral response induced by immunization, serum levels of antigen-specific IgG, IgG1, and IgG2c were measured by enzyme-linked immunosorbent assay (ELISA). High-binding microplates (Maxisorp, Nunc, Denmark) were coated overnight at 4 °C with a peptide pool composed of five synthetic epitopes derived from SARS-CoV-2 structural proteins. Each peptide was used at a concentration of 1 μg/mL per well, diluted in 0.1 molar bicarbonate-carbonate buffer (pH 9.0). After coating, wells were washed three times with phosphate-buffered saline containing 0.05% Tween-20 (PBS-T) and then blocked for 2 hours at 37 °C with PBS supplemented with 5% skimmed milk.

Serum samples from non-vaccinated and vaccinated animals were diluted in an incubation buffer (PBS-T containing 3% fetal bovine serum) and added to the plates in a volume of 100 microliters per well. Plates were incubated for 1 hour at 37 °C in a humidified atmosphere with 5% CO_2_. After additional washes with PBS-T, 100 microliters of horseradish peroxidase-conjugated secondary antibodies against mouse IgG (1:2500), IgG1 (1:5000), or IgG2c (1:20000) (Sigma Chemical Co., USA) were added to each well and incubated for another hour under the same conditions.

Following the final wash step, the substrate solution containing 1 mg/mL tetramethylbenzidine (TMB) and 4 µL of hydrogen peroxide (H_2_O_2_) in an acidic buffer (pH 5.0) was added and incubated in the dark at room temperature. After 10 min, the enzymatic reaction was stopped by adding 100 µL of 2 N sulfuric acid (H_2_SO₄), and absorbance was measured at 450 nm using a microplate reader.

### 2.13 *In silico* construction of multiepitope chimeric protein and evaluation of antigenic, allergenic, and physicochemical properties

Following immunogenic validation, five SARS-CoV-2 epitopes were assembled into multiepitope constructs, with β-defensin and PADRE sequences at the N-terminus joined by a rigid EAAAK linker. Three versions differing by linker type between epitopes were designed: rigid (EAAAK), flexible (GGGGGG), and protease-cleavable (PLGLWA).

The ProtParam tool from the Biopython library was used to assess physicochemical properties; constructs with instability index >40 were discarded. Antigenicity was predicted by VaxiJen v2.0 (https://www.ddg-pharmfac.net/vaxijen/), constructs with antigenicity <0.5 were discarded (27) and allergenicity by AllerCatPro v2.0 (https://allercatpro.bii.a-star.edu.sg) (28). Only stable, antigenic, and non-allergenic constructs were retained.

### 2.14 Molecular modeling, molecular dynamics, and molecular docking

3D models were generated using the AlphaFold3 server (https://www.alphafold.ebi.ac.uk/), applying default parameters to obtain high-confidence structural predictions (29). Molecular dynamics simulations (60 ns) were conducted in GROMACS with OPLS-AA/L force field, each construct was placed in a cubic unit cell with a 1.0 nm margin, solvated with water molecules, and neutralized by the addition of Na^+^ and Cl⁻ ions. Energy minimization was followed by equilibration under NVT and NPT ensembles for 100 ps each, maintaining a temperature of 300 K and pressure of 1 bar. Root mean square deviation (RMSD) analyses were used to assess structural fluctuations over time (30). Molecular docking with human TLR2 (PDB 2Z7X), TLR4 (3FXI), and TLR8 (5AWA) was performed via ClusPro (https://cluspro.bu.edu) (31). Binding interactions were analyzed for immune receptor engagement.

### 2.15 *In silico* immune simulation

Immune responses were simulated using C-ImmSim (https://kraken.iac.rm.cnr.it/C-IMMSIM/) under a triple-dose protocol (seed 12345, simulation volume 10), with injections administered at time steps 0, 100, and 200, corresponding to days 0, 30, and 60, respectively. Humoral (IgM, IgG1, IgG2, total Ig) and cellular cytokine profiles (IFN-γ, IL-2, IL-4, IL-10, TNF-α) were predicted to evaluate the immunogenic potential of constructs (32).

## 3 Results

### 3.1 Immune epitope mapping by dot blot serological assay

To investigate SARS-CoV-2 immunoreactivity, we assessed the IgG antibody response to five synthetic linear peptides derived from structural proteins of the virus: P1 (envelope protein), P2 (membrane glycoprotein), P3 (nucleocapsid), P4 and P5 (surface glycoprotein, Spike). These epitopes were previously identified through *in silico* immunoinformatic analysis (14).

**Figure 1:**
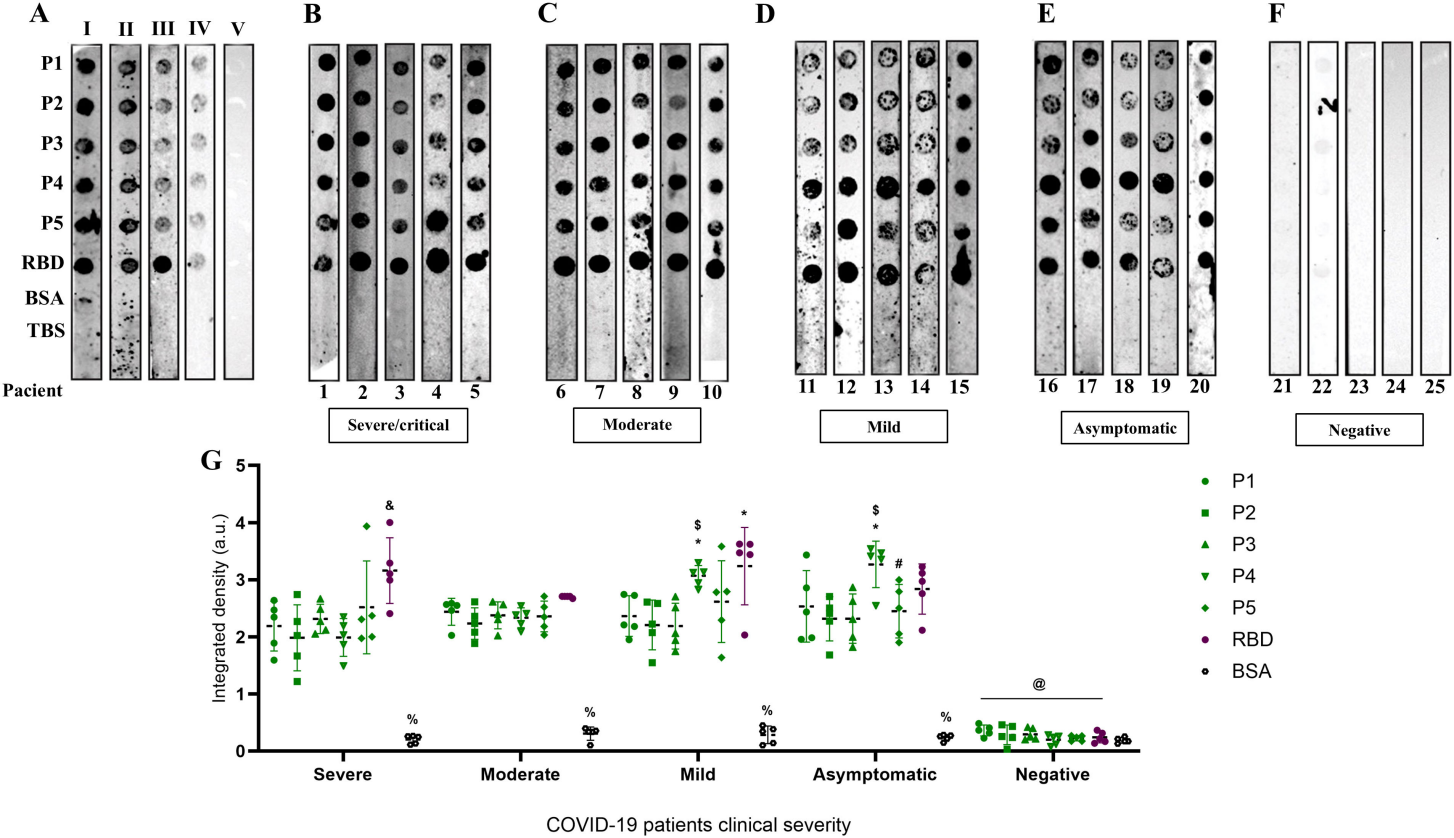
Detection and quantification of IgG antibodies against SARS-CoV-2 linear peptides by dot blot. **(A)** Limit of detection of the five linear peptides (P1–P5; 1 μg of each per membrane) determined by two-fold serial dilution of a serum sample from a COVID-19 patient with severe illness. Roman numerals I to V correspond to serum dilutions of 1:2,500, 1:5,000, 1:10,000, 1:20,000, and 1:40,000, respectively. **(B–E)** Representative dot blot images showing the immunoreactivity of peptides P1–P5 (1 μg each) with serum samples from patients with **(B)** severe/critical, **(C)** moderate, **(D)** mild symptoms, and **(E)** asymptomatic SARS-CoV-2 infection (n = 5 per group). **(F)** Representative dot blot image of sera collected prior to the COVID-19 pandemic (n = 5), used as negative controls. Recombinant SARS-CoV-2 receptor-binding domain (RBD) protein (0.5 μg) and bovine serum albumin (BSA; 0.5 μg) were included as positive and negative controls, respectively. **(G)** Densitometric analysis of dot blot assays performed using sera diluted 1:10,000. Each data point represents an individual serum sample (biological replicate). Signal intensities are expressed as arbitrary units (a.u.) and presented as mean ± SEM. Statistical analysis was performed using one-way ANOVA followed by multiple-comparison testing within each clinical severity group. Statistical significance is indicated in the graph as follows: *p < 0.05 related to P1, P2 and P3; **^#^**p < 0.05 related to P4; **^&^**p < 0.05 related to P1–P4; **^%^**p < 0.05 related to P1–P5 and RBD; **^$^**p < 0.05 related to P4 of Severe and Moderate groups; **^@^**p < 0.05 related to P1–P5 and RBD.

Dot blot assays were first optimized using serial dilutions (1:2,500 to 1:20,000) of serum from a patient with severe COVID-19. As shown in **Figure 1A**, each peptide (1 μg) and the recombinant receptor-binding domain (RBD, 0.5 μg) were spotted onto nitrocellulose membranes (0.22 μm) and efficiently recognized by the patient serum, confirming their antigenicity.

Subsequently, dot blot assays were conducted using serum samples from 25 individuals with confirmed SARS-CoV-2 infection, stratified according to clinical severity (severe, moderate, mild, or asymptomatic), as detailed in **Table 1**. All peptides were recognized by sera from infected patients (**Figures 1B–E**), regardless of disease severity, whereas pre-pandemic control sera showed no detectable reactivity (**Figure 1F**).

**Table 1:** Clinical spectrum of the patients with SARS-CoV-2 infection enrolled in this study.

Patient	Clinical severity	Symptoms	Coexisting disorders
1	Severe/critical	dry cough, dyspnea, headache and myalgia	diabetes, hypertension and obesity
2		dry cough, dyspnea, headache, fever and diarrhea	none
3		dry cough, dyspnea and fever	obesity
4		dry cough, dyspnea and fever	diabetes and obesity
5		fever, dyspnea and headache	none
6	Moderate	dry cough, fever, myalgia, asthenia	none
7		dry cough, dyspnea, fever and myalgia	HIV
8		dry cough dyspnea and fever	Deep vein thrombosis and pulmonary thromboembolism
9		dry cough and fever	obesity
10		dry cough dyspnea and fever	hypertension
11	Mild	dry cough, dyspnea and myalgia	none
12		dyspnea, fever and headache	
13		dry cough and dyspnea	
14		dry cough	
15		dry cough, dyspnea and fever	
16 - 20	Asymptomatic	none	none
21 - 25	Negative	none	none

Densitometric analysis of the dot blot signals (**Figure 1G**) confirmed that all five peptides (P1–P5) and RBD were recognized by sera from SARS-CoV-2–infected individuals across all clinical severity groups. In contrast, no reactivity against the irrelevant control protein (BSA) were observed, supporting assay specificity.

Within the Severe group, RBD showed significantly higher reactivity compared to peptides P1–P3. In the Mild group, both RBD and P4 displayed elevated signal intensity compared to several other peptides. In Asymptomatic individuals, P4 exhibited significantly higher reactivity compared to selected peptides. Similar patterns of peptide recognition were observed in the Moderate group.

Overall, although signal intensity varied among peptides within severity groups, recognition of all five peptides was consistently observed in infected individuals independent of clinical presentation.

### 3.2 Peptide stimulation combined with BCG activates the MAPK pathway and enhances inflammatory responses in restimulated BMDMs

**Figure 2:**
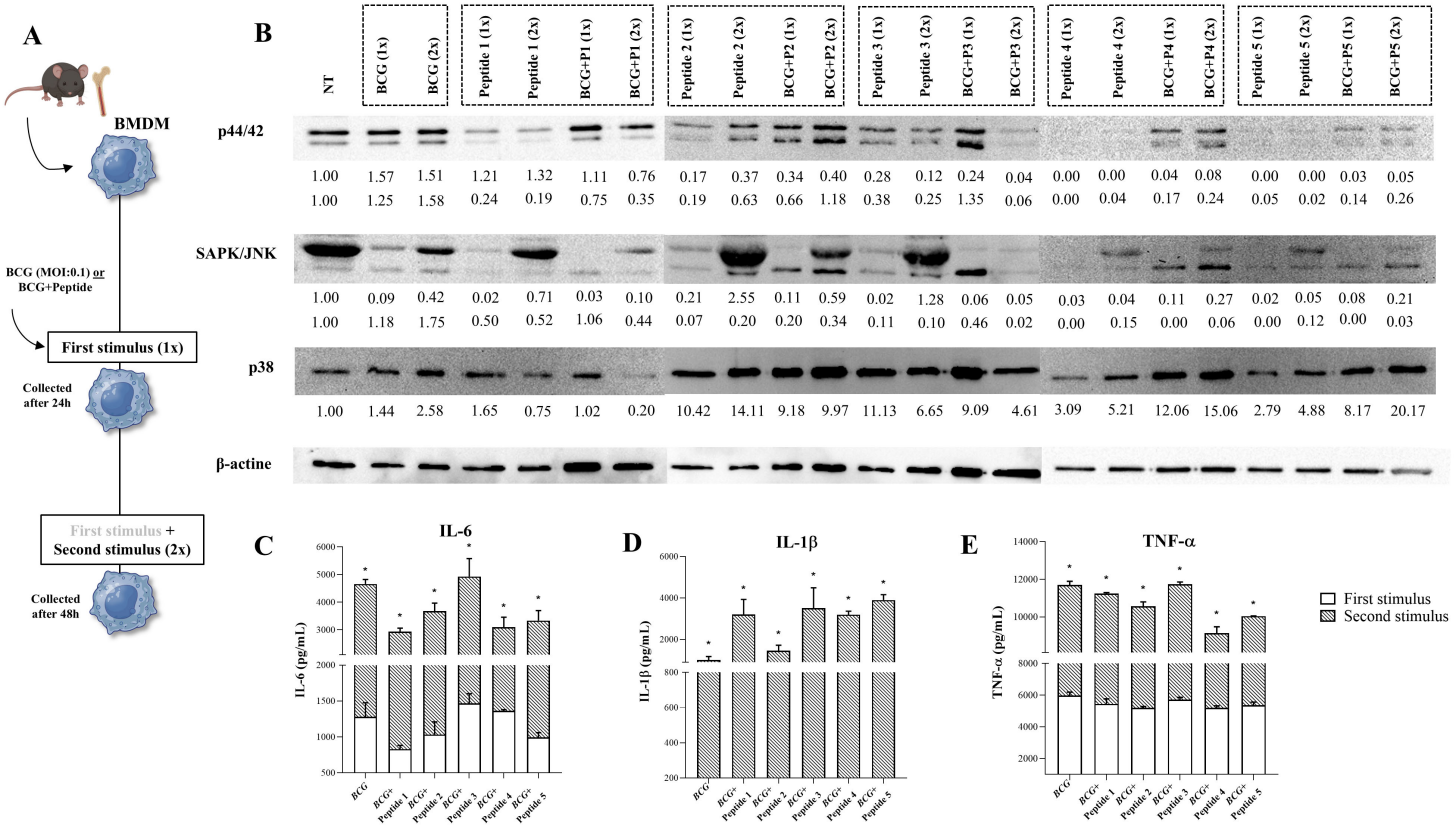
MAPK pathway activation and cytokine production in BMDMs stimulated with BCG and SARS-CoV-2 peptide-based formulations. **(A)** Schematic representation of the experimental design. BMDMs were stimulated with BCG (MOI 0.1), individual peptides (P1–P5), or combinations of BCG and peptides. Cells were harvested 24 h after the first stimulus (1×), or 48 h after the second stimulus (2×), and analyzed by Western blot or ELISA. **(B)** Western blot analysis of MAPK signaling proteins (p44/42, SAPK/JNK, and p38) following primary or secondary stimulation. β-actin was used as a loading control. Densitometric values below the bands represent normalized signal intensities relative to the untreated control (NT). **(C–E)** Quantification of IL-6 **(C)**, IL-1β **(D)**, and TNF-α **(E)** levels in culture supernatants after stimulation, determined by ELISA. Bars represent mean ± Standard Error of the Mean (n = 3, tested in triplicate). Statistical comparisons between first (1×) and second (2×) stimulation were performed using paired Student’s t-test. *p < 0.05 was considered statistically significant.

As illustrated in **Figure 2A**, bone marrow-derived macrophages (BMDMs) were obtained from mice and subjected to a primary stimulation with BCG (MOI 0.1), or combinations of BCG with peptides. After 24 hours, the cells were washed, resting without any stimulus for 72 hours, and subjected to a second stimulation under the same conditions. Samples were then collected after 48 hours for analysis of MAPK pathway activation by Western blot and cytokine production by ELISA.

Restimulation with formulations containing BCG and peptides resulted in a more robust activation of the MAPK pathway, particularly through the phosphorylation of the p38 protein (**Figure 2B**). Peptides P2, P3, P4, and P5 promoted a marked increase in p38 phosphorylation, both individually and in combination with BCG, with levels higher than those observed in the BCG-only and untreated groups. In contrast, ERK1/2 (p44/p42) phosphorylation showed reduced intensity in groups stimulated with peptides alone or in combination, suggesting selective modulation of the MAPK pathway.

Overall, restimulation induced greater p38 phosphorylation compared to the primary stimulation, indicating a profile compatible with innate trained immunity (20). Activation of other kinases, such as JNK and SAPK, was more modest, although occasional increases were observed in the P2 and P3 groups after the second stimulus.

IL-6, IL-1β, and TNF-α production was evaluated by ELISA (**Figures 2C–E**) and compared between the first and second stimulation phases were performed. Restimulation with formulations containing BCG and peptides led to a statistically significant increase in cytokine secretion compared to single stimulation (p < 0.05).

These data indicate that the formulation containing BCG in combination with specific peptides can selectively modulate elements of the MAPK pathway — with emphasis on p38 phosphorylation — and amplify the inflammatory response after restimulation, supporting its potential to induce a trained immunity phenotype.

### 3.3 BCG combined with SARS-CoV-2 peptides induces elevated IgG subclasses and circulating IL-6, TNF-α, and IFN-γ

**Figure 3:**
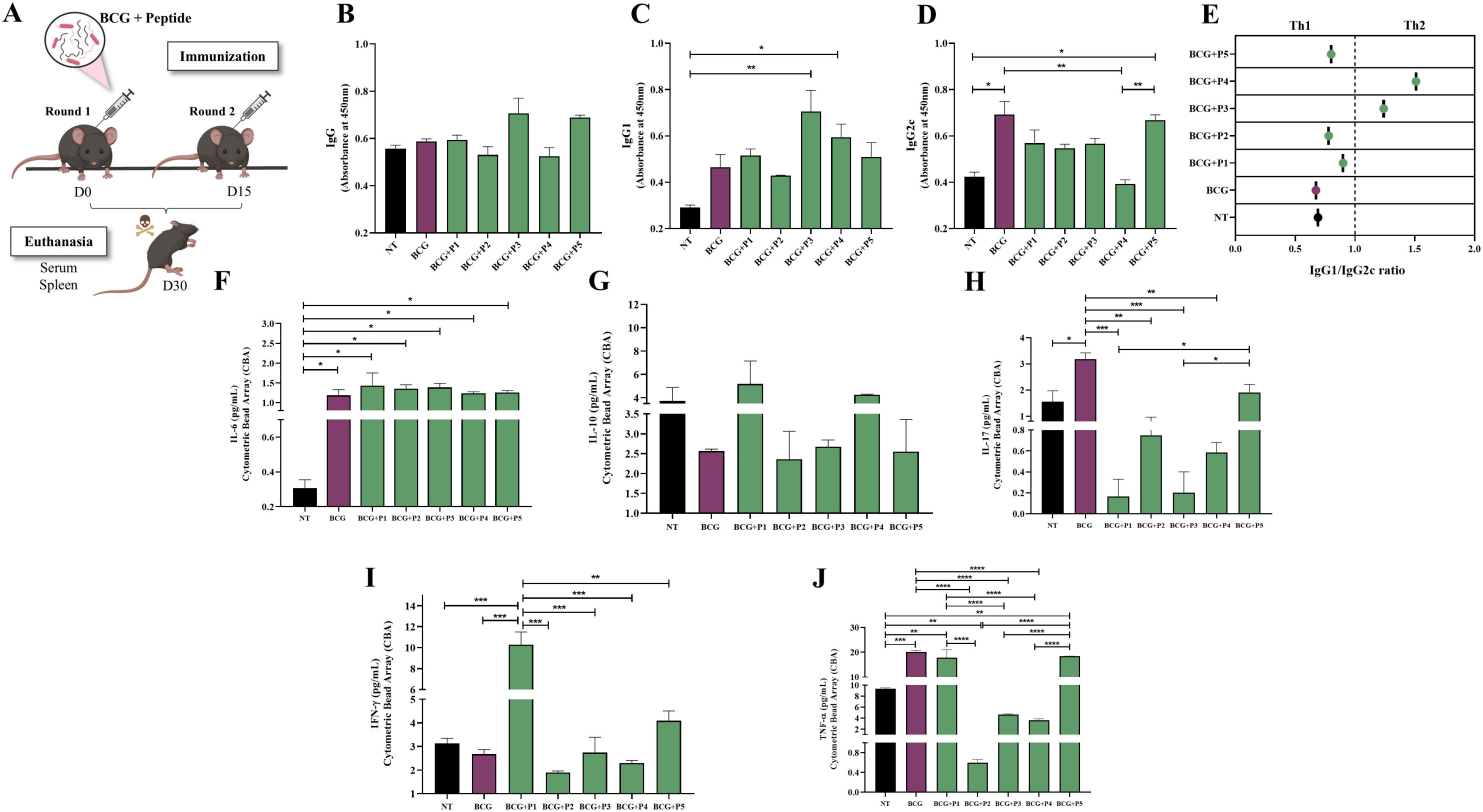
Immunization with BCG and SARS-CoV-2 peptides modulates the humoral and systemic cytokine responses in C57BL/6 mice. **(A)** Experimental design. Mice received intramuscular immunizations on days 0 and 15 with BCG or BCG combined with peptides (P1–P5). On day 30, serum and spleen samples were collected for analysis. **(B–D)** Evaluation of the humoral response by ELISA: total IgG **(B)**, IgG1 **(C)**, and IgG2c **(D)** levels in serum. **(E)** IgG1/IgG2c ratio as an indicator of Th1/Th2 polarization. Values <1 suggest a Th1-biased response, while values >1 indicate Th2 predominance. **(F–J)** Cytokine levels in serum are evaluated by CBA: IL-6 **(F)**, IL-10 **(G)**, IL-17 **(H)**, IFN-γ **(I)**, and TNF-α **(J)**. Bars represent mean ± SEM (n = 6). *p<0.05; **p<0.01; ***p<0.001; ****p<0.0001; one-way ANOVA followed by Tukey’s multiple comparisons test.

As illustrated in **Figure 3A**, C57BL/6 mice were immunized intramuscularly at two time points: day 0 (first dose) and day 15 (booster). Experimental groups included untreated animals (NT), BCG, and BCG combined with five different synthetic peptides (P1 to P5). On day 30, animals were euthanized for serum and spleen collection.

The induction of the humoral response was evaluated by quantifying IgG, IgG1, and IgG2c in the serum (**Figures 3B–D**). Although the BCG+P3 group showed a trend toward increased total IgG production, no statistically significant differences were observed among the groups (**Figure 3B**). In contrast, IgG1 levels (**Figure 3C**) were significantly increased in the BCG+P3 and BCG+P4 groups compared to NT, with BCG+P3 showing the highest levels of this immunoglobulin. The IgG2c analysis (**Figure 3D**) revealed a significant increase in the BCG and BCG+P5 groups compared to NT. Notably, the BCG+P4 group showed a significant reduction in IgG2c levels compared to both the BCG and BCG+P5 groups.

The IgG1/IgG2c ratio was evaluated as an indicator of Th1/Th2 polarization (**Figure 3E**). The NT (0.69), BCG (0.67), BCG+P2 (0.78), and BCG+P5 (0.80) groups showed ratios below 1, suggesting a Th1 profile. The BCG+P1 group had a ratio close to 1 (0.90), consistent with a balanced response. In contrast, the BCG+P3 (1.24) and BCG+P4 (1.51) groups showed a predominance of the Th2 profile. Suggesting a balanced Th1 and Th2 adaptive cellular response by epitopes during BCG presence as adjuvant.

Systemic cytokine production was evaluated in the serum by CBA (**Figure 3F–J**). For IL-6 (**Figure 3F**), all groups showed a significant increase compared to NT. IL-10 secretion (**Figure 3G**) showed no statistically significant differences among the groups. For IL-17 (**Figure 3H**), the BCG group exhibited significantly higher levels than NT. The BCG+P1 and BCG+P3 groups showed reduced levels compared to BCG+P5. Additionally, the BCG group showed higher IL-17 levels than all peptide-vaccinated groups, except BCG+P5, with significant differences. IFN-γ production was significantly elevated in the BCG+P1 group compared to all other groups, including NT, BCG, and all BCG+peptide groups (**Figure 3I**).

Finally, the TNF-α analysis (**Figure 3J**) showed that the BCG group had significantly higher levels than NT, as well as BCG+P2, BCG+P3, and BCG+P4. The BCG+P1 group also showed a significant increase compared to NT, P2, P3, and P4. The BCG+P5 group exhibited higher levels than NT, P2, and P3 and P4.

### 3.4 Splenocytes from mice immunized with BCG–peptide formulations exhibit increased IL-6, TNF-α, and IFN-γ secretion *ex vivo*

**Figure 4:**
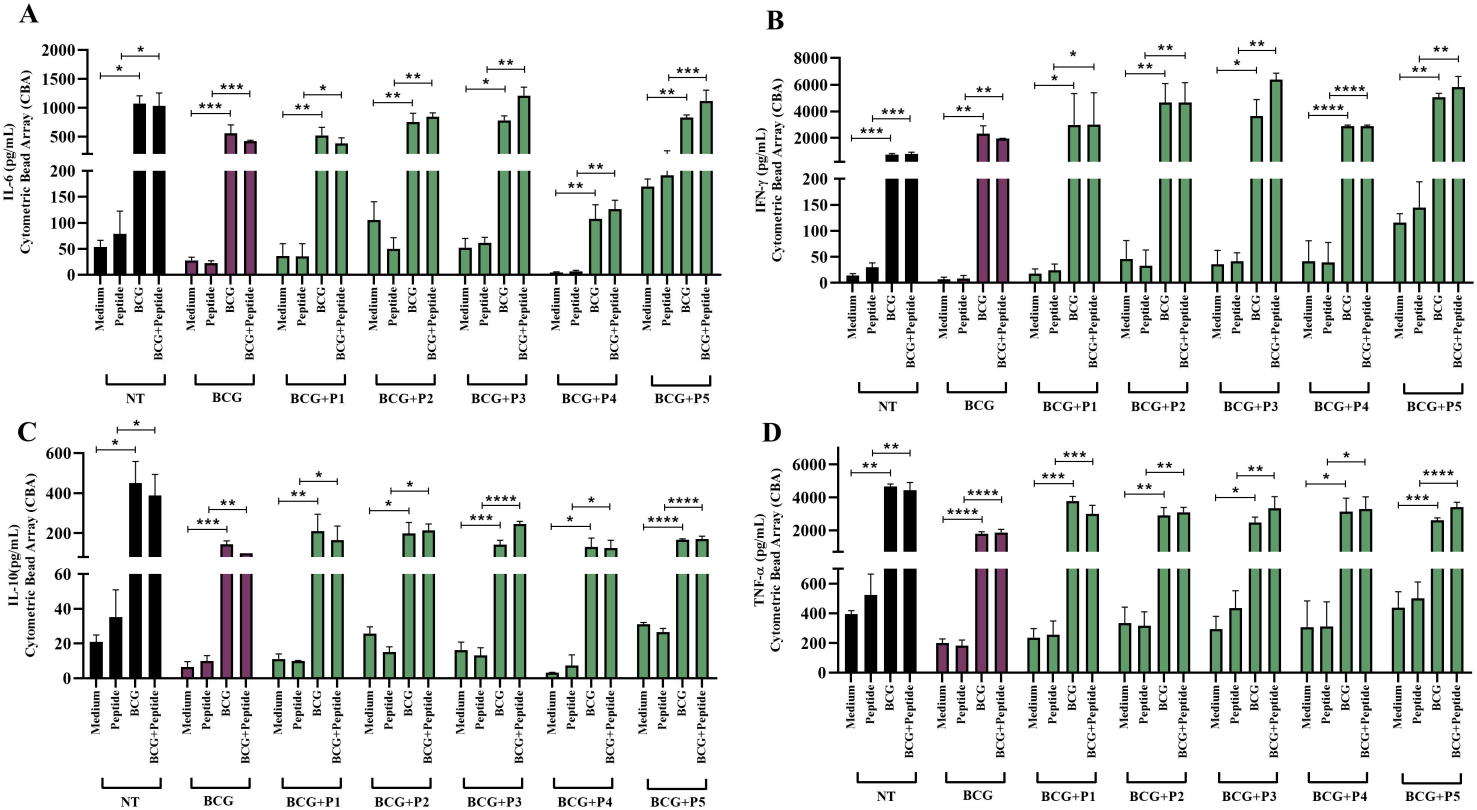
Cytokine production by splenocytes from immunized mice after in vitro stimulation. Splenocytes from C57BL/6 mice immunized with BCG or BCG–peptide formulations (P1–P5) were harvested on day 30 and stimulated *in vitro* for 24 h with medium, peptide, BCG, or BCG+peptide, according to the corresponding immunization group. **(A–D)** Cytokine levels in supernatants were measured by CBA: IL-6 **(A)**, IFN-γ **(B)**, IL-10 **(C)**, and TNF-α **(D)**. Each panel shows the response of splenocytes from non-treated (NT), BCG-immunized, and BCG+peptide-immunized mice under different stimulation conditions. Bars represent mean ± SEM (n = 6). *p<0.05; **p<0.01; ***p<0.001; ****p<0.0001; one-way ANOVA followed by Tukey’s post-test comparing stimuli within each immunization group.

To investigate the functional capacity of immunocompetent cells after immunization, splenocytes from mice were cultured for 24 hours in the presence of different stimuli (medium, peptide, BCG, or BCG+peptide), corresponding to the experimental groups (NT, BCG, BCG+P1 to P5). The concentrations of IL-6, IFN-γ, IL-10, and TNF-α in the supernatants were quantified by CBA (**Figures 4A–D**).

A consistent pattern was observed across all analyzed cytokines: cultures stimulated with medium or peptide alone showed low production levels, with no statistically significant differences between them. In contrast, stimulation with BCG or BCG+peptide induced significantly increased responses. These results suggest that BCG acts as a potent adjuvant, promoting activation of previously immunized splenocytes.

In the cytokine analysis (**Figure 4**), the NT group showed a significant increase in IL-6 production (**Figure 4A**) upon stimulation with BCG and BCG+peptide compared to medium and peptide stimulation. Similar results were observed for the vaccinated groups (BCG, BCG+P1, BCG+P2, BCG+P3, BCG+P4, and BCG+P5), which also exhibited higher cytokine levels when stimulated with BCG or BCG+peptide versus medium and peptide. This pattern was consistent for IFN-γ (**Figure 4B**), IL-10 (**Figure 4C**), and TNF-α (**Figure 4D**), with all groups showing markedly increased secretion in response to BCG and BCG+peptide stimuli compared to their respective controls.

### 3.5 Design and *in silico* evaluation of multiepitope vaccine candidates incorporating SARS-CoV-2 structural epitopes

**Figure 5:**
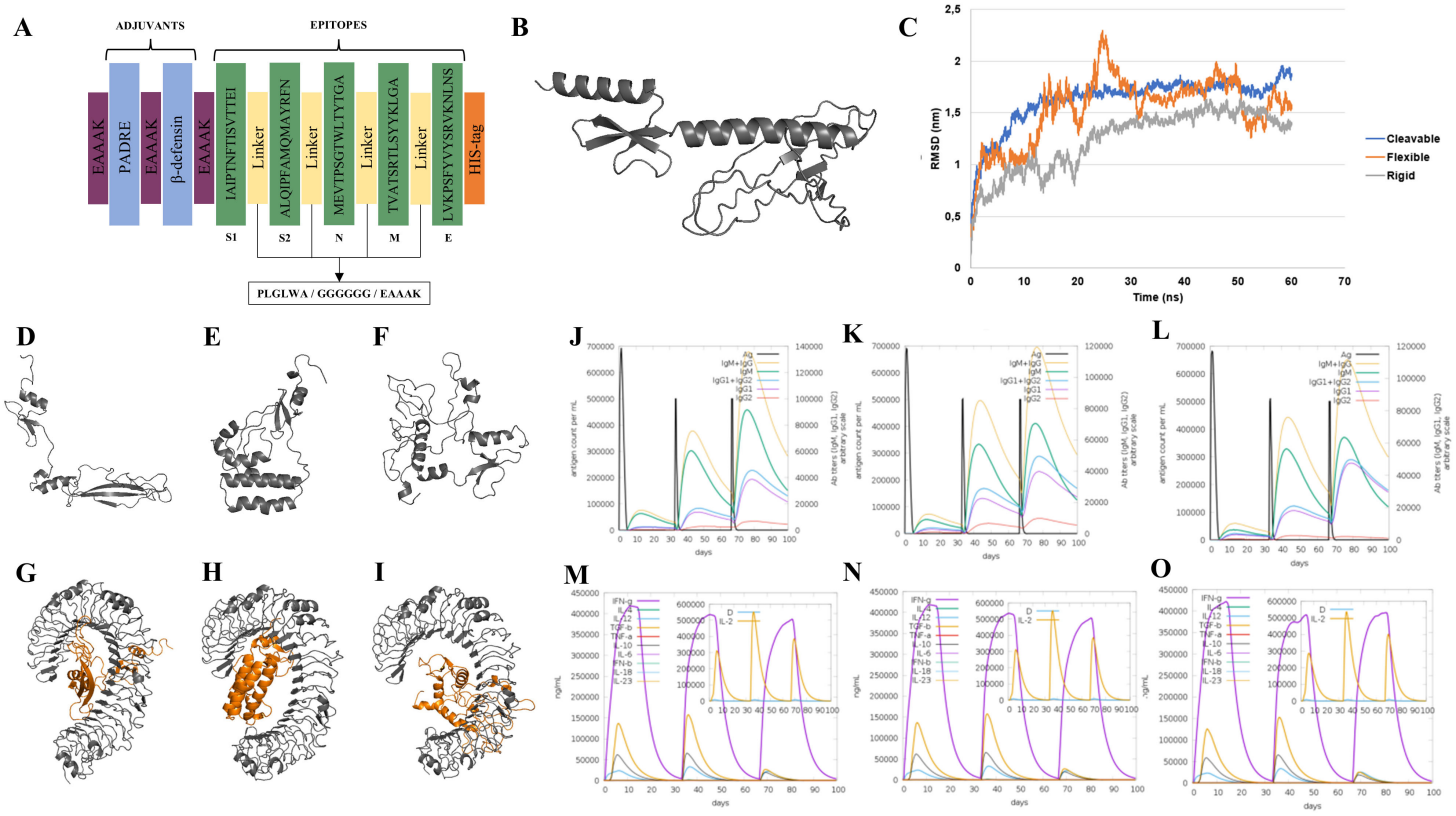
*In silico* design and evaluation of multiepitope vaccine constructs against SARS-CoV-2. **(A)** Schematic representation of the three chimeric proteins constructed with five conserved T and B cell epitopes from SARS-CoV-2 structural proteins (S, N, M, and E), combined with immunostimulatory adjuvants (β-defensin, PADRE, and EAAAK linker) and different inter-epitope linkers: cleavable (PLGLWA), flexible (GGGGGG), or rigid (EAAAK). **(B)** Representative 3D structure of the designed chimeric protein modeled by homology using AlphaFold. **(C)** RMSD values obtained during 60 ns molecular dynamics simulations using GROMACS, indicating structural stability of the rigid, flexible, and cleavable constructs. **(D–F)** Final conformations of the flexible **(D)**, rigid **(E)**, and cleavable **(F)** proteins after simulation. **(G–I)** Molecular docking results of the chimeric proteins with the TLR4 receptor using ClusPro: flexible **(G)**, rigid **(H)**, and cleavable **(I)**. Docked proteins are shown in orange. **(J–L)** Humoral immune response simulation via C-ImmSim for flexible **(J)**, rigid **(K)**, and cleavable **(L)** constructs, showing antibody titers (IgM, IgG1, IgG2, and IgG3) over time. **(M–O)** Cellular immune response simulation for flexible **(M)**, rigid **(N)**, and cleavable **(O)** constructs, highlighting IFN-γ and other cytokines (IL-2, IL-10, IL-12, TGF-β) following a three-dose schedule on days 0, 30, and 60. All constructs showed strong immunogenicity with no excessive inflammatory response.

Based on the previously identified in silico epitopes P1 to P5 (14), validated in this study through *in vitro* and *in vivo* approaches, three multiepitope vaccine candidates were designed. The topological order of the epitopes in the virus (from surface to core) was preserved, and only the type of linker used was varied: flexible (GGGGGG), rigid (EAAAK), or cleavable by metalloproteases (PLGLWA). To enhance immunogenicity, two immunomodulatory adjuvants were incorporated at the N-terminal: β-defensin, which stimulates antigen-presenting cells (APCs), and PADRE, which favors MHC class II presentation. Both were connected by rigid linkers, resulting in three constructs named flexible, rigid, and cleavable proteins (**Figures 5A, B**).

The three variants were evaluated for antigenicity, allergenicity, and physicochemical properties. All showed antigenicity values above the predictive threshold (>0.5) according to the VaxiJen 2.0 server: 0.635 (flexible), 0.609 (rigid), and 0.593 (cleavable). None demonstrated allergenic potential, as determined by AllerCatPro 2.0. Physicochemical parameters obtained via ProtParam indicated structural stability (instability index <40), high solubility in *Escherichia coli* (values >0.85 in SoluProt), basic nature (pI ~9.9), and half-lives compatible with heterologous expression. A comparative summary of the main *in silico* characteristics and performance metrics of the three constructs is presented in **Table 2**. Among the constructs, the flexible variant exhibited the most promising profile for biotechnological applications.

**Table 2:** Comparative *in silico* evaluation of the three multiepitope vaccine constructs.

Parameter	Flexible	Rigid	Cleavable
Linker	GGGGGG	EAAAK	PLGLWA
VaxiJen score	0.635	0.609	0.593
Allergenicity	Non-allergen	Non-allergen	Non-allergen
Instability index	< 40 (stable)	< 40 (stable)	< 40 (stable)
Solubility (SoluProt)	> 0.85 (soluble)	> 0.85 (soluble)	> 0.85 (soluble)
Theoretical pI	~9.9	~9.9	~9.9
RMSD range (nm)	0.5–2.3	0.5–1.4	0.5–1.9
Structural stability	Highest flexibility	Highest stability	Intermediate stability
TLR4 docking energy (kcal/mol)	-1050.8	-1018.3	-1018.8
Predicted peak antibody titer	~140,000	~120,000	~120,000
Predicted IFN-γ production	~450,000	~450,000	~450,000
Danger index	none	none	none
Overall characteristic	Strongest humoral response	Greatest structural stability	Balanced profile

Three-dimensional modeling of the proteins was performed using AlphaFold3, and the structures were visualized in PyMOL (**Figure 5B**). Structural predictions showed high confidence in regions corresponding to adjuvants and linkers, but lower accuracy in epitope regions, likely due to a lack of similar crystallographic models. The constructs were then submitted to 60 ns molecular dynamics simulations using the GROMACS 2019.3 package with the OPLS-AA/L force field. RMSD analysis showed that the rigid protein had the lowest structural deviation (0.5–1.4 nm), followed by the cleavable (0.5–1.9 nm) and the flexible proteins (0.5–2.3 nm), suggesting greater stability of the rigid protein (**Figure 5C**). The final structures obtained after 60 ns are shown in **Figure 5D** (flexible), **Figure 5E** (rigid), and **Figure 5F** (cleavable).

Molecular interaction between the chimeric proteins and the TLR4 receptor was evaluated by molecular docking using ClusPro. All variants exhibited favorable binding energy values, with the flexible protein showing the most negative score (–1050.8 kcal/mol; **Figure 5G**), followed by the cleavable (–1018.8 kcal/mol; **Figure 5I**) and the rigid proteins (–1018.3 kcal/mol; **Figure 5H**). Detailed analyses revealed the formation of multiple non-covalent interactions in critical receptor regions, indicating good affinity and possible activation of the innate signaling pathway.

Immune response simulation predicted robust humoral and cellular responses for all constructs after three doses. The flexible protein reached the highest levels (~140,000; **Figure 5J**), while the rigid (**Figure 5K**) and cleavable (**Figure 5L**) proteins reached values around 120,000. All constructs promoted high levels of IFN-γ (~450,000 units), as well as the production of cytokines such as TGF-β, IL-10, IL-12, and IL-2, with zero values for the danger index (**Figures 5M–O**). The immune response elicited was balanced, with no signs of inflammatory exacerbation. Overall, the flexible protein stood out for the intensity of the humoral response, while the rigid protein showed greater structural stability.

## 4 Discussion

The impact caused by the emerging SARS-CoV-2 virus highlighted the need for effective and adaptable vaccines, particularly those capable of remaining functional against mutations that give rise to variants of concern (VOCs) (1, 4, 10). These variations, resulting from rapid viral evolution, are mainly concentrated in the RBD region of the Spike protein and may lead to reduced immunity over time in the currently available vaccines, which are based on messenger RNA, viral vectors, or attenuated/inactivated viruses (8–11). In this context, it becomes necessary to develop vaccine candidates that prioritize conserved regions and other proteins capable of inducing an adequate immune response without being significantly affected by mutations (14–18).

In this scenario, subunit vaccines, such as peptide-based ones, emerge as a promising alternative, since they allow the selection of conserved regions and can be used in patients belonging to at-risk groups, such as immunocompromised individuals, pregnant women, and the elderly (14–18). Our group previously identified, through in silico analyses, conserved epitopes in structural proteins of SARS-CoV-2 present in different VOCs, aiming at the development of multi-epitope vaccines capable of inducing specific immune responses (14). Since the previous study was conducted exclusively in silico, experimental validation was required. In this study, we demonstrated that all predicted peptides were recognized by antibodies present in the serum of convalescent patients, confirming the translational relevance of computational predictions and the natural presentation of these epitopes during infection. The recognition of these epitopes in patients with different clinical presentations of the disease makes them viable not only as vaccine targets but also for the development of specific immunodiagnostic tests against various viral variants (33–35).

Despite being recognized, the epitopes present an important limitation compared to vaccines that use attenuated viruses: low immunogenicity (14, 36, 37). Therefore, the use of adjuvants that promote more robust immune responses becomes essential in the development of multi-epitope vaccine candidates (36, 37). A promising example is *Bacillus Calmette-Guérin* (BCG), which can act both as an adjuvant and as a delivery platform (19–22, 33). Studies have shown that BCG is capable of inducing enhanced innate and adaptive immune responses (19–22, 33). Moreover, recent reports indicate that BCG vaccination can elicit cross-reactive immune responses and enhance protection against SARS-CoV-2 through mechanisms of trained immunity and adjuvant activity rather than direct peptide homology (38–40). The present study reinforces this potential, demonstrating that conserved epitopes of SARS-CoV-2 structural proteins, when combined with BCG, induce robust innate and adaptive immune responses. These findings are consistent with recent evidence highlighting BCG as a potent adjuvant and immunomodulatory platform in the development of COVID-19 vaccines (38–40).

Dot blot analyses confirmed the antigenicity of synthetic peptides derived from the E, M, N, and S proteins. This result supports studies showing that conserved epitopes outside the Spike protein can sustain broad immunoreactivity even in the face of emerging viral mutations (33). In particular, epitopes derived from the nucleoprotein (P3) showed high immunogenic potential, consistent with previous reports that nucleoprotein antigens are recognized in different clinical presentations of COVID-19 and can serve as reliable targets for next-generation vaccines (33, 35).

**Figure 6:**
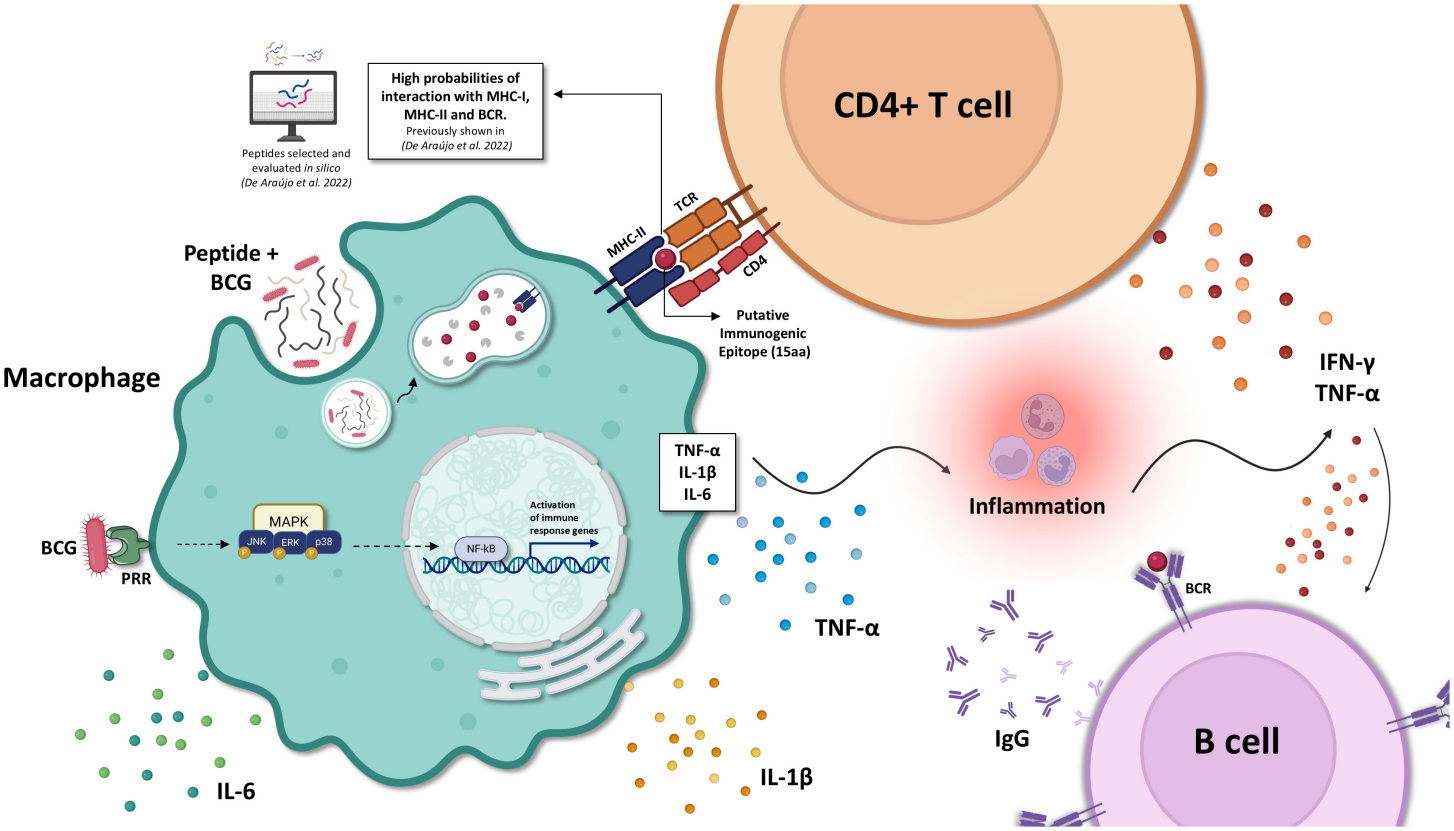
Proposed mechanism of action for the BCG–peptide formulation inducing innate training and adaptive immune activation. Synthetic peptides previously selected in silico (De Araújo et al., 2022) and formulated with BCG are phagocytosed by macrophages, where they activate pattern recognition receptors (PRRs), leading to stimulation of the MAPK signaling cascade (notably via p38, ERK, and JNK) and subsequent NF-κB-mediated transcription of proinflammatory genes. This process results in increased secretion of inflammatory cytokines such as IL-6, IL-1β, and TNF-α. The immunogenic peptides are processed and presented via MHC-II, interacting with CD4^+^ T cells and promoting their activation. This interaction enhances the production of cytokines such as IFN-γ and TNF-α, contributing to a proinflammatory microenvironment. B cells are activated through BCR recognition and T cell help, leading to IgG secretion. Together, these responses establish a profile compatible with trained innate immunity and potentiate adaptive responses. This model illustrates how BCG serves as an adjuvant platform that enhances the immunogenicity of peptide antigens, ultimately bridging innate and adaptive arms of the immune system and supporting the development of novel peptide-based vaccine strategies.

Regarding innate immune modulation, we observed that BCG–peptide formulations promoted selective phosphorylation of p38 MAPK and increased secretion of IL-6, IL-1β, and TNF-α after restimulation, supporting a trained immunity phenotype (**Figure 6**). This profile is consistent with studies in humans showing that BCG vaccination induces strong cytokine responses to heterologous challenges, including marked increases in TNF-α and IL-6 (38–40). The ability of BCG to modulate the MAPK pathway and amplify inflammatory responses suggests that the peptides acquire an additional layer of immunogenicity when administered together with BCG.

At the adaptive level, BCG combined with peptides induced differential IgG subclass responses. We observed increased IgG1 in the BCG+P3 and BCG+P4 groups, whereas IgG2c was preferentially induced in BCG+P5, reflecting Th2- and Th1-polarized responses, respectively. Although total IgG levels did not differ significantly among groups, the observed modulation of IgG subclasses indicates that the formulations influenced the quality and polarization of the humoral response rather than simply increasing overall antibody production. These data resemble experimental vaccines based on BCG, such as BCG: CoVac, where a single dose induced high titers of neutralizing IgG and robust Th1 polarization, conferring sterilizing immunity against viral challenge in hACE2 mice (22). Similarly, recombinant BCG constructs expressing N and S antigens induced neutralizing antibodies (IgG2c) and IFN-γ secretion, resulting in lower viral loads and reduced lung pathology in murine models (**Figure 6**) (39).

Cytokine analysis further reinforced the adjuvant effect of BCG. Elevated levels of IFN-γ, TNF-α, and IL-6 were observed in animals immunized with peptide–BCG, in agreement with experiments in which the co-administration of BCG with inactivated SARS-CoV-2 vaccines in hamsters increased antibody titers, suppressed pulmonary viral load, and reduced lesion severity compared to the vaccine alone (**Figure 6**) (40). Importantly, splenocyte assays showed that restimulation with BCG or BCG+peptide markedly enhanced cytokine production, indicating a potentiated immune activation beyond the classical immune response induced by BCG.

Considering the responses elicited by individual epitopes, we hypothesize that the construction of a chimeric protein by concatenating these epitopes may represent an additional promising strategy. Our in silico analyses for this hypothesis revealed that multi-epitope constructs incorporating these peptides exhibited favorable characteristics of antigenicity, stability, and interaction with TLR4, in addition to immunological simulations predicting strong IgG and IFN-γ responses (14–18). This dual strategy, combining epitope design with BCG’s ability to induce balanced immunity, positions such constructs as promising vaccine candidates to overcome the limitations of Spike protein-based formulations alone (33).

Taken together, our results, combined with recent experimental evidence, support the use of BCG both as a delivery system and as an adjuvant for conserved SARS-CoV-2 epitopes. By simultaneously engaging potential trained innate immunity and the adaptive response, this approach may provide broader and longer-lasting protection against SARS-CoV-2 variants.

However, this study presents limitations that must be considered. It represents an initial preclinical experimental model without direct viral challenge evaluation, which restricts extrapolation to protective efficacy in humans. Sexual dimorphism in immune responses has been widely described in both coronavirus infections and vaccination settings, including differences in disease severity, viral clearance, inflammatory responses, and cytokine profiles (41, 42). Therefore, restricting the analysis to female animals may limit the generalizability of our findings. Future studies incorporating both sexes and different age groups, as well as viral challenge models, will be essential to provide a more comprehensive evaluation of this vaccine strategy. Furthermore, although *in silico* and *in vivo* analyses point to promising results, clinical trials will still be indispensable to validate the safety and efficacy of this vaccine strategy.

## 5 Conclusion

This study demonstrated that conserved epitopes from SARS-CoV-2 structural proteins are naturally recognized by antibodies from convalescent patients and can be potentiated when combined with BCG, leading to activation of the MAPK pathway, compatible with trained innate immunity, and robust adaptive responses in mice. The observed modulation of IgG subclasses and cytokine production supports the role of BCG as a versatile adjuvant or delivery platform, capable of bridging innate and adaptive immunity.

Furthermore, *in silico* analyses confirmed that multiepitope constructs incorporating these peptides exhibit favorable antigenicity, stability, and immune simulation profiles, with the flexible and rigid variants emerging as particularly promising prototypes. Together, these findings provide proof-of-concept for the development of next-generation vaccines that combine conserved epitopes with BCG-based strategies to achieve broader and more durable protection against SARS-CoV-2 variants. Nevertheless, further preclinical studies with viral challenge models and subsequent clinical validation will be essential to confirm the translational potential of this approach.

## Data Availability

The original contributions presented in the study are included in the article/**Supplementary Material**. Further inquiries can be directed to the corresponding author.
