# Supplementary material for: Bacillus Calmette-Guérin as adjuvant platform enhances immunogenicity of conserved epitopes from structural proteins of SARS-CoV-2
Source: Front Immunol. 2026 Jul 16;17:1775087. doi: 10.3389/fimmu.2026.1775087 (PMC13422184; doi:10.3389/fimmu.2026.1775087)
Supplement: Supplementary file 1 [file Table1.docx]

Supplementary Material

# Supplementary Figures


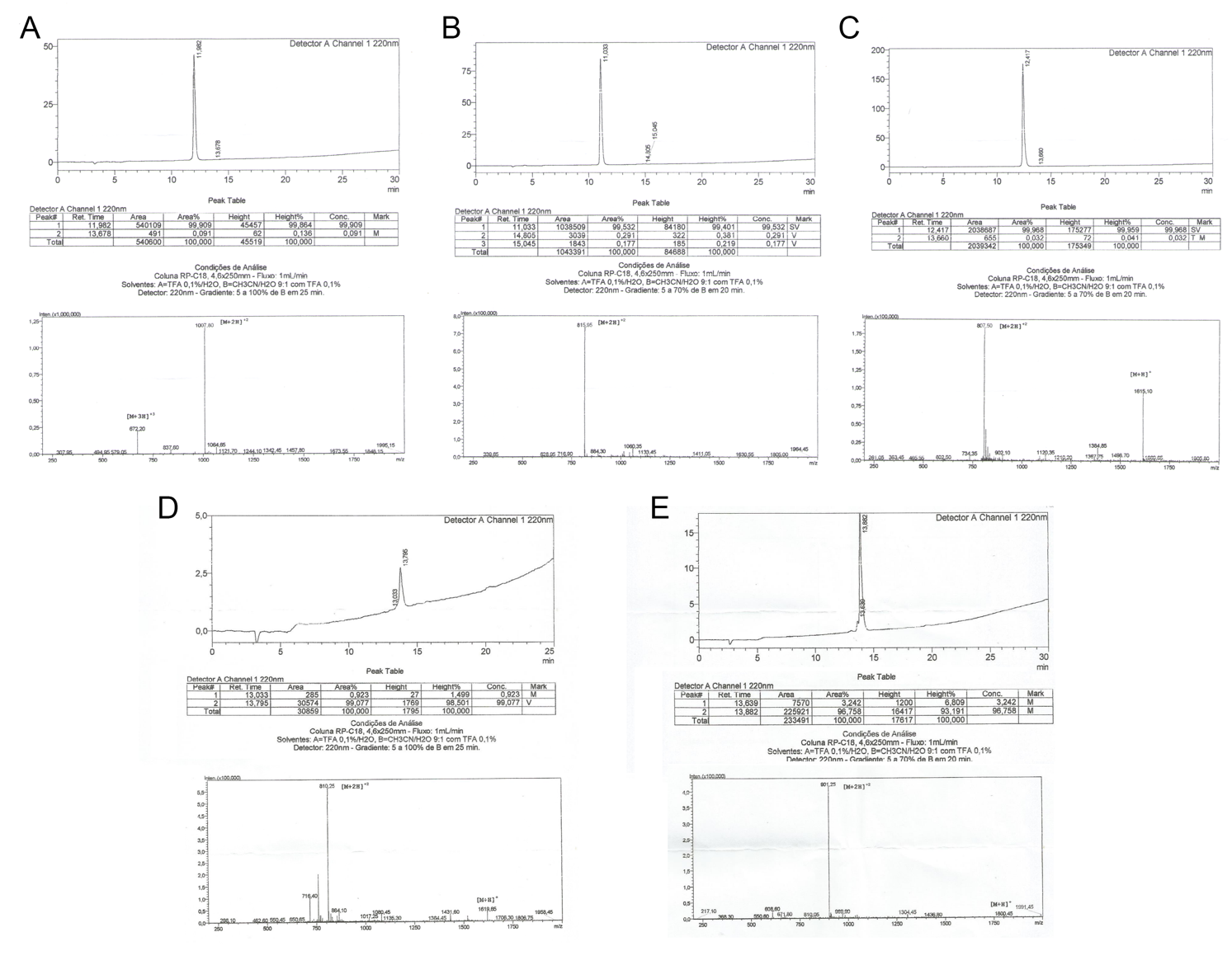


**Supplementary Figure 1.** Analytical characterization of the synthetic peptides corresponding to immunodominant epitopes of SARS-CoV-2 structural proteins. Each peptide was synthesized by solid-phase peptide synthesis and purified by reverse-phase HPLC. The identity of each peptide was confirmed by mass spectrometry. (A) Envelope protein epitope (P1). (B) Membrane glycoprotein epitope (P2). (C) Nucleocapsid phosphoprotein epitope (P3). (D) Spike glycoprotein epitope 1 (P4). (E) Spike glycoprotein epitope 2 (P5). HPLC chromatograms are shown on top and the corresponding mass spectra below each panel.
